# Measurement of the tilt of a moving domain wall shows precession-free dynamics in compensated ferrimagnets

**DOI:** 10.1038/s41598-020-73049-5

**Published:** 2020-10-01

**Authors:** E. Haltz, J. Sampaio, S. Krishnia, L. Berges, R. Weil, A. Mougin

**Affiliations:** Université Paris-Saclay, CNRS, Laboratoire de Physique des Solides, 91405 Orsay, France

**Keywords:** Magnetic properties and materials, Spintronics

## Abstract

One fundamental obstacle to efficient ferromagnetic spintronics is magnetic precession, which intrinsically limits the dynamics of magnetic textures. We experimentally demonstrate that this precession vanishes when the net angular momentum is compensated in domain walls driven by spin–orbit torque in a ferrimagnetic GdFeCo/Pt track. We use transverse in-plane fields to provide a robust and parameter-free measurement of the domain wall internal magnetisation angle, demonstrating that, at the angular compensation, the DW tilt is zero, and thus the magnetic precession that caused it is suppressed. Our results highlight the mechanism of faster and more efficient dynamics in materials with multiple spin lattices and vanishing net angular momentum, promising for high-speed, low-power spintronic applications.

## Introduction

**Figure 1:**
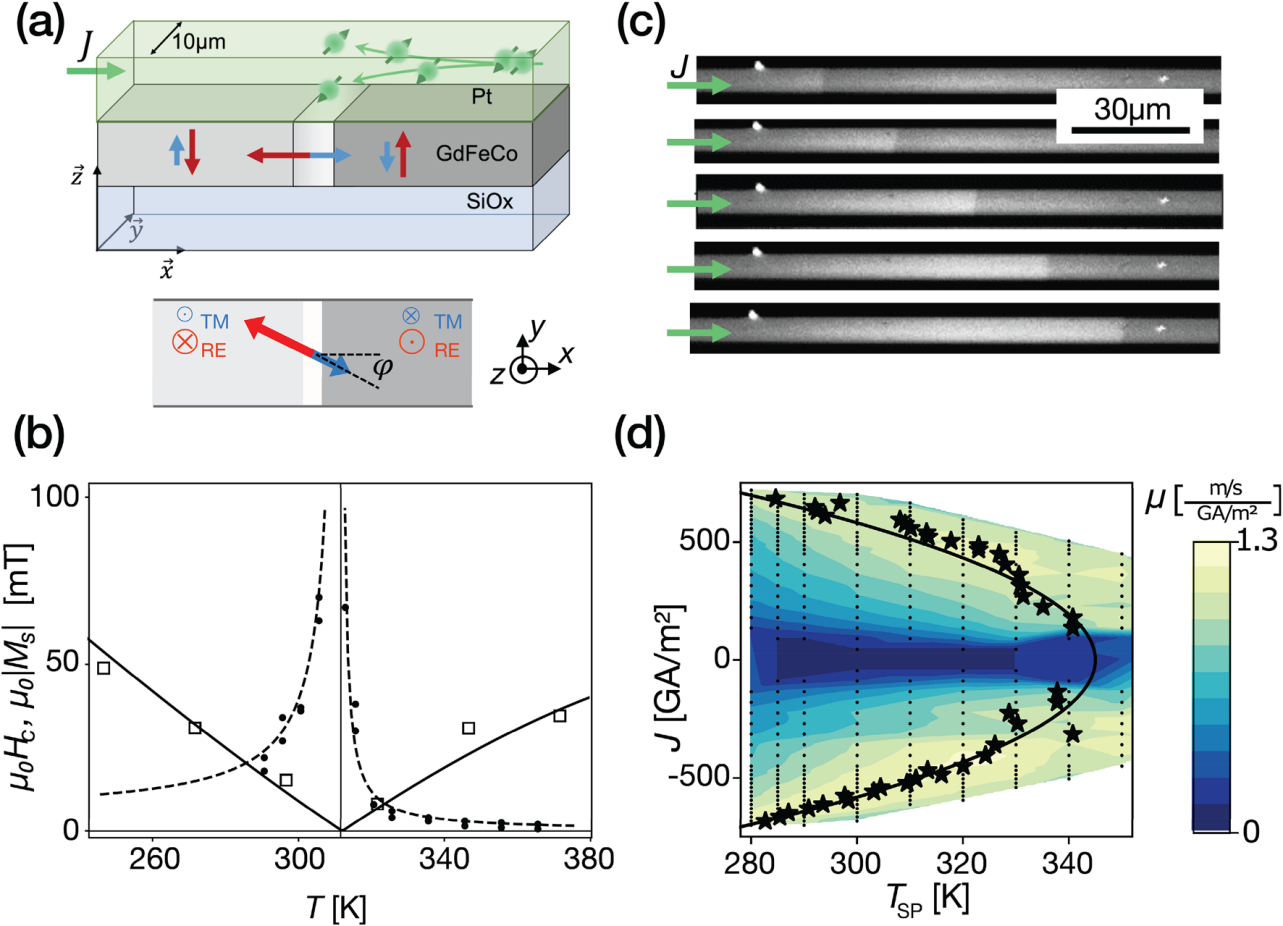
GdFeCo/Pt sample properties and SOT-driven DW propagation in tracks. **(a)** Sketch of the track containing a SOT-driven DW and of the magnetisation of the two sublattices (in red and blue) below *T*_MC_. The size of the arrows represents their relative magnitude. The grayscale corresponds to the domain Kerr contrast while the DW is depicted in white. The angle of the DW magnetisation is given by *φ*. **(b)** Measured net magnetisation *M*_S_ (squares) of the virgin film and coercivity *H*_C_ (dots) of the patterned track versus temperature *T*. The *M*_S_ points were shifted by − 31 K to account for migration of Gd during patterning^12^. The continuous line is the result of the mean-field model (see suppl.). **(c)** Kerr images of a DW driven by 300 GA/m^2^, 25 ns current pulses in at temperature set-point of *T*_*SP*_ = 300 K. **(d)** (*J,T*_*sp*_) colour-plot of measured mobilities (black points). Stars mark the peak of mobility *µ* versus *T*_*SP*_ (interpolated; see suppl.). The solid line is a quadratic fit of the maximum mobilities, with 345 K = *T*_SP_ + *k J*^2^ (*k* = 1.3 10^–4^ K/(GA m^−2^)^2^).

In magnetic materials, the exchange interaction aligns the magnetic moments producing ferromagnetic or antiferromagnetic orders. Even if ferromagnets have numerous applications in spintronics, two effects limit the development of higher-density and faster devices. Firstly, the stray fields couple adjacent magnetic textures and limit their density. Secondly, the magnetic precession changes the internal magnetisation of moving textures, resulting in e.g. a continuous precession in field- or spin-transfer-torque-driven DWs above Walker threshold^1–3^, in a steady-state internal angle in SOT-driven DWs^4,5^, or in the topological deflection of skyrmions^6–9^. All these effects limit the texture’s velocity. Antiferromagnetic order leads to faster dynamics and robustness against spurious fields, and is emerging as a new paradigm for spintronics^10^. However, perfect antiferromagnets with exactly compensated magnetic sub-lattices are hard to probe and manipulate, and therefore have been rarely studied or used in applications. Rare Earth-Transition Metal (RETM) ferrimagnetic alloys allow to benefit from both antiferromagnetic-like dynamics and ferromagnetic-like spintronic properties. Indeed, they have two antiferromagnetically-coupled sublattices, corresponding roughly to the RE and TM moments, and their spin transport is carried mainly by the TM sub-lattice^11^. Furthermore, RETM thin films can exhibit perpendicular magnetic anisotropy, are conductors, and present large spin transport polarization and spin torques, even when integrated in complex stacks^12^. Additionally, their magnetic properties can be controlled by changing either their composition or temperature, as described by the mean-field theory^11^. For a given composition, they can exhibit two characteristic temperatures: the angular momentum compensation temperature (*T*_AC_) and the magnetic compensation temperature (*T*_MC_), for which the net angular momentum or the net magnetisation (*M*_S_) are respectively zero (Fig. 1b). Interestingly, due to the different Landé factors of RE and TM, these two temperatures are different (with *T*_MC_ < *T*_AC_ for GdFeCo). At *T*_MC_, the magnetostatic response vanishes (as observed in the divergence of the coercivity, anisotropy field, …). In contrast, at *T*_AC_ the dynamics is affected. Although these effects are challenging to evidence, the singular and promising behaviour of RETM at *T*_AC_ was observed in current-induced switching^13^, magnetic resonance^14^, and time-resolved laser pump-probe measurements^15,16^. In very recent reports the signature of the dynamics at *T*_AC_ was assigned to a DW mobility peak, under field^17,18^, under current by spin–orbit torques (SOT)^19–22^, or by spin transfer torque^23^. However, even if this mobility peak is a signature of angular compensation, it is affected by the strong sensitivity of DW propagation to Joule heating and pinning^24,25^. Furthermore, none of the latter experiments gives a direct access to the internal DW magnetisation angle that is an intrinsic signature of the magnetisation precession. In this paper, we use a robust measurement of the variation of the DW velocity with a transverse bias field to determine the DW internal magnetisation angle across the compensation temperatures, and we show that there is no DW magnetisation tilt, and therefore no magnetic precession, at the angular moment compensation.

## Results

DWs driven by SOT have been observed in thin ferrimagnetic films with a heavy-metal adjacent layer, like Pt, which induces three main interfacial effects: perpendicular anisotropy, Dzyaloshinskii-Moriya exchange interaction (DMI), and vertical spin current generated by the spin Hall effect (SHE) (Fig. 1a). Such systems present chiral Néel DWs^26,27^, which is the configuration for which the SOT DW driving is most effective (Fig. 1a)^4^.

To investigate SOT-driven DW dynamics in RETM, a 10 µm-wide track of amorphous Gd_.4_(Fe_.85_Co_.15_)_0.6_ (5 nm) capped with Pt (7 nm) with perpendicular magnetic anisotropy was fabricated (Fig. 1a). *M*s(*T*) was measured by SQUID magnetometry (Fig. 1b). Due to the migration of Gd during patterning^12^ the *M*_S_ values have changed. By measuring the *T*_MC_ pre- and post-patterning, we corrected this *M*_S_ temperature shift of − 31 K. A relatively low and temperature-dependent magnetisation has been measured as expected^12^. The magnetic compensation temperature where the net magnetisation vanishes is clearly visible. Transport measurements of the extraordinary Hall effect (EHE) versus field were made on 5 μm wide crosses at different temperatures for both in-plane and out-of-plane magnetic fields. The *T*_MC_ of the track, 312 K, was determined by measuring the coercivity divergence (Fig. 1b). It diverges at *T*_MC_ as the applied field produces opposite and balanced effects on the two compensated sub-networks. The magnitude of the SOT was determined with the second harmonic Hall voltage method^28,29^.

DW velocity measurements were performed using a Kerr microscope with a controlled temperature sample holder (at temperature set-point *T*_SP_). 25 ns pulses of current density *J* were applied in the track containing a DW. After each pulse, a Kerr image is recorded. The DWs move against the electron flow, which is compatible with SOT-driving of chiral Néel DWs with the same relative sign of DMI and SHE as the one found in ferromagnetic Pt/Co^4,30^ and which rules out any significant spin-transfer torque^25^. The linearity of the DW displacement with the pulse number and duration (see suppl.) allows the robust determination of the propagation velocity *v*. The magnitude of DMI was determined by analysing the DW velocity driven by electrical current under an in-plane field (*H*_x_) collinear to the current^31^ (see suppl.). Since in perpendicularly-magnetised tracks SOT induced propagation depends on the DW in-plane magnetisation, the reversal of the DW propagation induced by the in-plane field also validates the SOT-driven mechanism.

High DW velocities (> 700 m/s; see velocity curves in suppl.) are observed for low *J* (~ 600 GA/m^2^), as previously reported in similar alloys^19,20^. The DW mobility *µ* = *v*/*J* exhibits a peak that depends on the *T*_*SP*_ and the current density *J*. Figure 1d shows measured mobilities in a (*J,T*_*sp*_) colour-plot, and for each *J* the maximum *µ* is marked with a star. The coordinates of the maxima *µ* follow $T - T_{SP} \propto J^{2}$ (solid line in Fig. 1c)*,* which suggests that they all occur at a single track temperature *T* = 345 K. In ferromagnets, models predict that the SOT-driven DW steady-state velocity follows

1 $$ v/J \propto \cos \left( \varphi \right) $$

where $\varphi$ is the angle of the internal DW magnetisation^4^. The angle *φ* is determined by the balance between DMI, which favours the Néel configuration (*φ* = 0), and the precession induced by SOT, which increases $\left| \varphi \right|$. In ferrimagnets, it is expected that the precession depends on temperature and vanishes at *T*_AC_ with a peak in velocity (See suppl.). If the effects of pinning and Joule heating are neglected, it is possible to attribute the observed mobility peak with minimal $\left| \varphi \right|$, and it can be deduced that the temperature of the maxima is *T*_AC_ (345 K according to the fit in Fig. 1c), as previously done in Refs.^19,20^.

In order to overcome these limitations, we propose a new method based on the application of a transverse field *H*_Y_ that reveals the internal magnetic dynamics of the DW. It provides both a qualitative and quantitative evaluation of $\varphi$, including its sign, across both compensation points, without requiring any additional sample magnetic parameters. Simultaneously, it determines the Joule heating amplitude.

**Figure 2:**
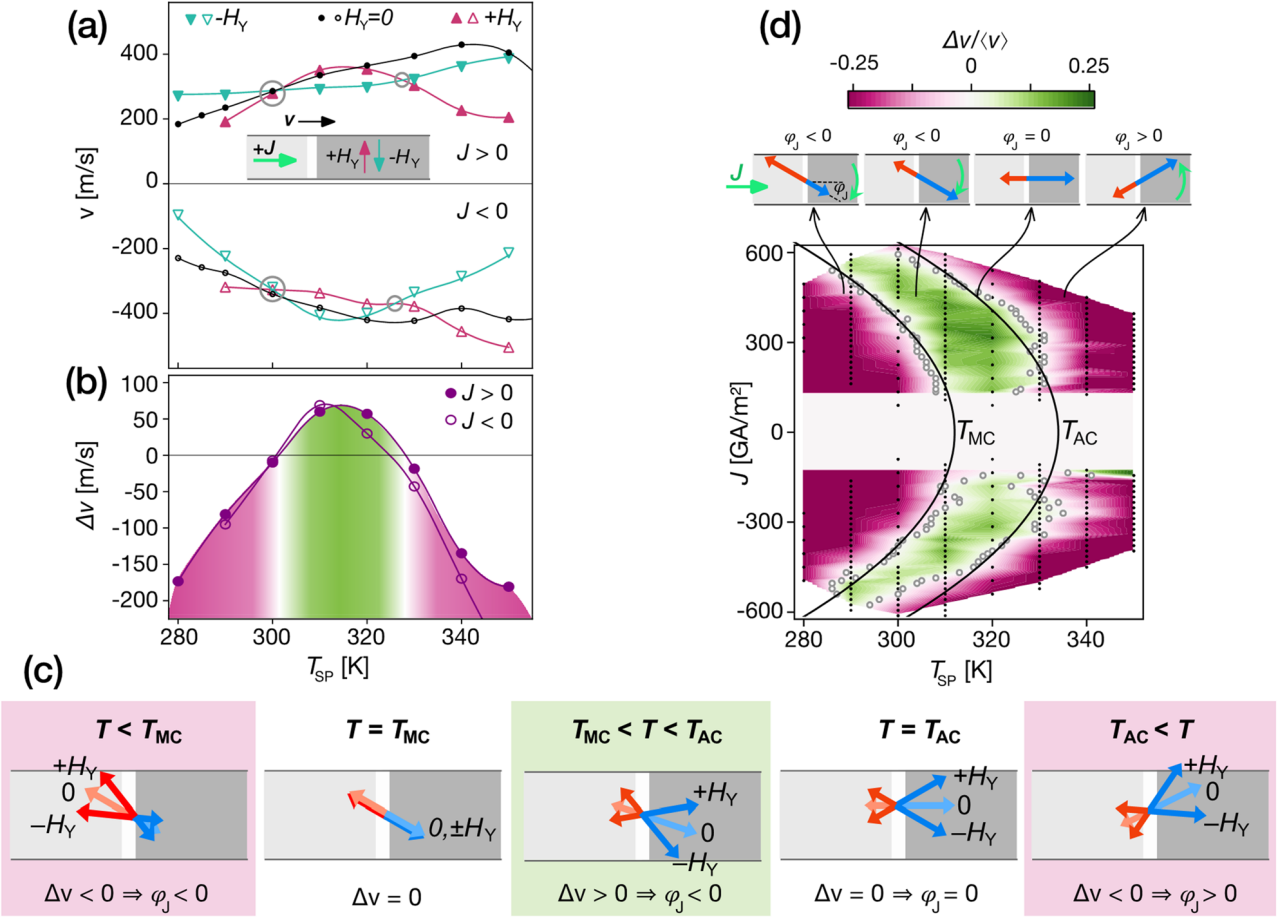
SOT-driven DW under *H*_Y_: determination of the internal DW dynamics, *T*_MC_ and *T*_AC_. **(a)** Measured DW velocity *v* versus sample holder set-point temperature *T*_SP_ with µ_0_*H*_Y_ =  ± 90 mT or 0 mT, for *J* =  ± 360 GA/m^2^. **(b)** Velocity difference Δ*v*(*T*_SP_) ≡ *v*(*J*, + *H*_Y_) − *v*(*J*,-*H*_Y_) from (a). **(c)** Diagram of the sublattice orientations in a SOT-driven DW under *H*_Y_ across compensation points. The red and blue arrows correspond to RE and TM, respectively. **(d)** Colour-plot of Δ*v/‹v›*(*J*, *T*_SP_). Black points correspond to measurements. Grey circles correspond to crossing points where Δ*v* = 0 (see (**a**)). The black lines are parabolic fits of these crossing points (*T* = *T*_SP_ + *k J*^2^, *k* = 0.8 10^–4^ K/(GA/m^2^)^2^, *T*_MC_ = 312 K, *T*_AC_ = 334 K). Above, the action of the SOT on the internal DW angle $\varphi_{J}$ is sketched, to illustrate the sense of rotation of $\varphi_{J}$ without field. Throughout, pink and green backgrounds mark the sign of Δ*v*.

We measured the DW velocity *v* versus *T*_SP_ with an applied in-plane field *H*_Y_ perpendicular to the current flow (see inset of Fig. 2a). Figure 2a shows the velocity *v*(*T*_SP_, *H*_Y_) without field (µ_0_*H*_Y_ = 0) and with two opposite fields (µ_0_*H*_Y_ =  ± 90 mT) for positive and negative current density (*J* =  ± 360 GA/m^2^). Whatever the *H*_Y_ field, the DW moves along the current direction. Two crossing points, at *T*_SP_ = 300 K and *T*_SP_ = 328 K, are observed where *v*(*T*_SP_, + *H*_Y_) = *v*(*T*_SP_, − *H*_Y_). On the first crossing point, the velocity without field is the same as with field, *v*(300 K, 0) = *v*(300 K, ± *H*_Y_), while on the second crossing point the velocity without field is larger than with field, *v*(328 K, 0) > *v*(328 K, ± *H*_Y_). The crossing points are more readily distinguished by plotting Δ*v*(*T*_SP_) ≡ *v*(*T*_SP_, + *H*_Y_) − *v*(*T*_SP_, − *H*_Y_), shown in Fig. 2b, and are the same for both current polarities.

To understand the effect of *H*_Y_ on SOT-driven DWs in ferrimagnets, we first consider the well-understood ferromagnetic case. The *H*_Y_ couples to the internal magnetisation of the DW and changes the equilibrium *φ*. As *v*/*J ∝ *cos(*φ*) (Eq. 1), if + *H*_Y_ rotates *φ* closer to Néel configuration, it will increase the velocity. The sign of Δ*v* shows whether + *H*_Y_ rotates *φ* closer to or farther from the Néel configuration compared to − *H*_Y_. In particular, a positive Δ*v* means that + *H*_Y_ and *J* have opposite contributions to *φ* (and Δ*v* < 0 means + *H*_Y_ and *J* push *φ* in the same direction)*.* Since the sign of the effect of *H*_Y_ is known, we can deduce the sign of the *φ* angle without field, that we note *φ*_J_.

In the RETM ferrimagnetic case, the DW velocity can still be described with the same model^32^. Since the spin current interacts mainly with the TM sub-lattice (^12^and references therein), *φ* in Eq. (1) corresponds to the DW angle of the TM sub-lattice (see Fig. 1a). The Zeeman contribution of *H*_Y_ depends now on the net magnetisation *M*_S_ = *M*_TM_ − *M*_RE_, which changes sign at *T*_MC_.

Figure 2c shows a sketch of the in-plane magnetisation of the RE and TM sublattices at the centre of the SOT-driven DW at different temperatures. At *T* < *T*_MC_, the RE sublattice is dominant (*M*_TM_ < *M*_RE_) and + *H*_Y_ rotates *φ* clockwise. At *T* = *T*_MC_, *M*_RE_ = *M*_TM_ and *H*_Y_ does not affect *φ* nor *v*, and *v*(*H*_Y_ = 0) = *v*(± *H*_Y_). Above *T*_MC_, *M*_TM_ > *M*_RE_ and the effect of external fields is reversed: + *H*_Y_ rotates *φ counter*clockwise.

In Fig. 2b, Δv < 0 below T_SP_ = 300 K, so we conclude that the current acts on φ in the same direction as + H_Y_, i.e. φ_J_ < 0. We observe that *T*_MC_ occurs at *T*_SP_ = 300 K, as v(H_Y_ = 0) = v(± H_Y_). At this point, it is not possible to determine the φ_J_. Above T_MC_, interestingly, the measured Δv crosses zero once more (*T*_SP_ = 328 K). Below this point, Δ*v* > 0 so φ_J_ < 0, and above it Δ*v* < 0 so φ_J_ > 0. At the crossing point, the current does not affect φ: φ_J_ = 0. The fact that the velocity with ± *H*_Y_ are smaller than without field confirms the symmetrical configuration shown in Fig. 2c with φ_J_ = 0 (see suppl. mat. for other values of *H*_Y_). The observed reversal of the direction of the precession, and the precession-free point, is characteristic of the angular compensation, T_AC_.

We measured this quantity for different current densities and the obtained behaviour is very similar. Figure 2d shows all measured Δ*v/*‹*v*› in a colour-plot as a function of *T*_SP_ and *J,* normalized by the average velocity with + *H*_Y_ and -*H*_Y_. This normalization removes the first-order dependence on |*J*| of Eq. (1) ($v \propto \cdot J \cdot \cos \varphi \left( J \right)$), as ${\Delta }v/v = 2\frac{{\cos \left( {\varphi_{{ + H_{Y} }} } \right) - \cos \left( {\varphi_{{ - H_{Y} }} } \right)}}{{\cos \left( {\varphi_{{ + H_{Y} }} } \right) + \cos \left( {\varphi_{{ - H_{Y} }} } \right)}}$ is independent of *J*, enabling the direct comparison of data for different current densities. Three regions can be observed with, successively, Δ*v* < 0, Δ*v* > 0 and Δ*v* < 0, separated by the two sets of crossing points. These crossing points depend on *J* but their difference is independent of *J* (see data in suppl.)*.* Indeed, both follow a Joule heating parabolic relation with same heating parameter (within 1%), which can be associated to the isothermal lines of *T*_MC_ = 312 K and *T*_AC_ = 334 K. These observations hold for different magnitudes of *H*_Y_ (see suppl.). Also, spurious external fields have low impact on the crossing points (see calculations in suppl.). Note that *T*_MC_ and *T*_AC_ are consistent with the previous measurements of *H*_C_(T) in Fig. 1b (*T*_MC_ = 312 K) and *µ*(*J*) in Fig. 1d (*T*_AC_ = 345 K). Furthermore, the measured values of Δ*v* are large (few hundreds of m/s), give directly the sense of precession of the magnetisation and show that the angle $\varphi$ of the moving DW changes sign and vanishes at *T*_AC_.

## Discussion

In ferromagnets, the angle *φ* can be described with the 1D model^4^, extended to include external magnetic fields:

2 $$ \varphi = \arctan \left( {\frac{\Delta }{D}\left( {\frac{{ \hbar \theta_{SHE} }}{2\,e t}\frac{ J}{\alpha } + \mu_{0} {\text{M}}_{s} {\text{H}}_{y} } \right)} \right) $$

where $\Delta$ is the DW width parameter, *D* is the DMI parameter, $\alpha$ is the Gilbert damping parameter, *ħ* is the reduced Planck constant, *e* is the fundamental charge, *θ*_SHE_ is the spin Hall angle of the Pt layer, and *t* is the magnetic film thickness. For a ferrimagnet, $\varphi$ corresponds to the DW angle of the TM sub-lattice (see Fig. 1a), *M*_S_ to the net magnetisation, and *α* is the effective Gilbert damping parameter. The observed reversal and vanishing of the precession dynamics (*φ*_*J*_ = 0) at *T*_AC_ is directly associated with a divergence and change of sign of this effective Gilbert damping parameter α(*T*) in Eq. (2), as described in Refs.^15,16,33^ (Note that in Ref.^17^ it is stated that the α parameter does not diverge at T_AC_. However, the authors refer to their new and distinct definition of α that is not the conventional Gilbert’s parameter. Gilbert’s α does diverge, as it is discussed briefly in Ref.^17^). Note that, even if α diverges and changes sign, the dissipation power, which is proportional to the product of α and the net angular momentum, remains finite and positive even across *T*_AC_. This effective parameter approach was successfully used to describe ferrimagnetic dynamics observations^15,16,20,34^.

**Figure 3:**
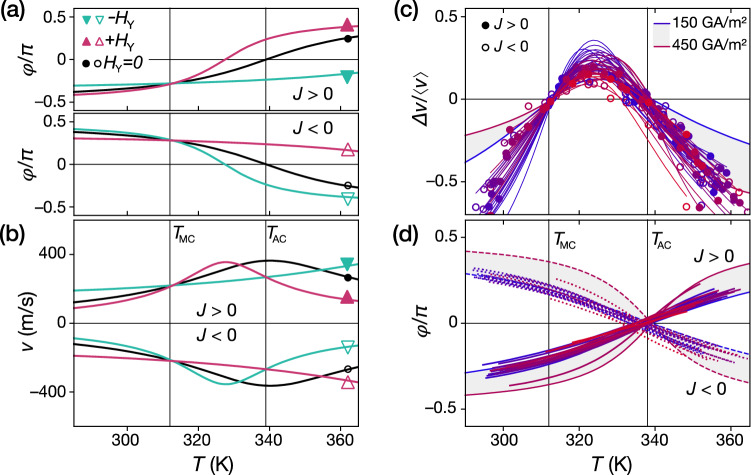
Determination of the internal DW angle. **(a, b)** Calculated *φ* and *v* according to Eqs. (1), (2), using the material parameters taken from measurements (see Fig. 1b,c and suppl.). µ_0_*H*_Y_ =  ± 90 mT, and *J* =  ± 360 GA/m^2^. (**c)** Experimental Δ*v/‹v›* for different current densities. The thin lines correspond to fits using a simplified version of Eq. (2) (see text). The data were horizontally shifted so that their first crossing points are juxtaposed at *T*_MC_ = 312 K. (**d)** Obtained *φ*_*J*_ from the fits in (c). The thick lines and envelopes in (c) and (d) correspond to the theoretical curves, obtained with the same parameters of (a) and (b).

Figure 3a,b show analytical calculations of the DW angle *φ* and related velocity *v* as a function of *T* and *H*_Y_. All material parameters were taken from measurements (see Fig. 1b,c and suppl.), except for effective damping parameter α(*T*) which was approximated by $\propto$ 1/(*T* − *T*_AC_) to account the expected divergence at *T*_AC_. All other quantities (Δ, *D, θ*_*SHE*_) are taken as constant in the narrow investigated range. We observe an excellent agreement between the theoretical curves and the experimental data in Fig. 2a. We can also verify the explanation given above (Fig. 2c): at *T*_MC_, the *φ* and *v* are the same for all *H*_Y_ and, at *T*_AC_, *φ* are opposite for + *H*_Y_ and − *H*_Y_*.*

All Δ*v/‹v›* are shown in the same graph versus *T* in Fig. 3c. Since we know that all the first crossing points occur at the same track temperature (*T*_MC_ = 312 K), we shifted all the curves in Fig. 3c so the crossing points overlap at *T*_MC_. Note that, for a given *J*, Δ*v/‹v›* is only a function of *φ*(+ *H*_y_*)* and *φ*(− *H*_y_), with no other parameters^28^. We use the approximation of $\alpha \propto 1/\left( {T - T_{AC} } \right)$ and $M_{S} \propto T - T_{MC}$ to get a simplified version of Eq. (2): $\varphi \left( {T, \pm H_{Y} } \right) = \arctan \left( {Ja\left( {T - T_{a} } \right) \pm H_{Y} b\left( {T - T_{b} } \right)} \right)$, that is used to fit the Δ*v/‹v›* points for each current density (thin lines in Figs. 3c,d). The first term corresponds to the current contribution, and should change sign at *T*_AC_, as the effective damping does, and the second term should change sign at *T*_MC_, like *M*_S_. The fitting indeed gives *T*_a_ = 336 ± 3 K ≈ *T*_AC_, *T*_b_ = 312 ± 2 K ≈ *T*_MC_ (and *a* = − (0.3 ± 0.1) 10^–3^ (K·GAm^−2^)^−1^ and *b* = − 0.04 ± 0.01 (K·T)^−1^). We plot the temperature evolution of *φ*_*J*_ in Fig. 3d, obtained directly from *a* and *T*_*a*_. Without field, the angle *φ*_*J*_ follows the sketch of Fig. 2c, and is in agreement with the theoretical curve of Fig. 3a. All data Δ*v/‹v› (*Fig. 3c) and the fitted *φ*_*J*_ (Fig. 3d) are in the envelope that corresponds to the theoretical curve for *J* between 150 and 450 GA/m^2^.

In GdFeCo/Pt with Néel DWs and DMI, we measured how the velocity of an SOT-driven DW changes with an in-plane transverse bias field *H*_y_. This bias field changes the internal angle of the magnetisation of the DW (*φ*_*J*_) that affects its velocity (v ~ cos *φ*_*J*_). By analysing the sign of the velocity difference with + *H*_y_ and—*H*_y_ (Δ*v*), it is possible to determine the sign of *φ*_*J*_. We found that there are two temperatures for which Δ*v* = *0* and we showed that they correspond to the *T*_MC_ and *T*_AC_. These measurements also reveal the vanishing of the tilt of the magnetisation at *T*_AC_ (*φ*_*J*_ = 0), which had been theoretically predicted but never directly observed. This novel approach determines precisely the magnitude and the sense of the DW tilt for a moving DW, which is a consequence of the magnetic precession of the spins through which the DW travels. This method gives *T*_MC_ and *T*_AC_ and is based on the intrinsic DW dynamics and so is unaffected by DW pinning. Finally, the velocity difference is easily observed (Δ*v* ≈ 100 m/s), independent of the Joule heating and does not require knowledge of material parameters.

The suppression of magnetic precession opens new perspectives for fast and energy-efficient spintronics using any angular-momentum-compensated multi-lattice material. It induces a maximum of SOT-driven DW mobility in a compensated RETM ferrimagnet, as we observed in agreement with previous reports^19–21^. Here, for the first time, direct experimental evidence is provided that the SOT-driven DW propagation is tilt-free and the DW remains Néel (*φ* = 0) in angular momentum compensated ferrimagnets. These dynamics in angular-momentum-compensated materials are also interesting for skyrmion dynamics, and it has been shown that it leads to efficient manipulations^35^, and vanishing topological deflection^22^.

## Materials and methods

### Sample deposition, fabrication

The film of amorphous GdFeCo(5 nm) capped with Pt(7 nm) was deposited by electron beam co-evaporation in ultrahigh vacuum on thermally-oxidised Si substrates. Details of the film growth and characterisation can be found in^12^. The tracks were patterned by e-beam lithography and hard-mask ion-beam etching.

### Characterisation of the magnetic properties

Transport measurements of the extraordinary Hall effect versus field were made on 5 μm crosses at different temperatures in a commercial QD PPMS. The magnitude of the SOT equivalent field *H*_DL_ was determined with the harmonic voltage method^28,29^. *M*_S_(*T*) was measured by SQUID magnetometry. The magnitude of DMI equivalent field *H*_DMI_ was determined by analysing the SOT-driven DW velocity with a field collinear to the current as in^31^.

### Kerr microscopy

Kerr microscopy experiments were performed using an adapted commercial Schafer Kerr microscope, with a temperature regulated sample holder. The DW velocity was measured by taking Kerr images before and after each of about ten current pulses (see Fig. 1c). The linearity of the DW displacement with the number of pulses and with the pulse duration allowed a reliable determination of the propagation velocity *v*.

### Analytical model of DW velocity under SOT and field

Equations (1) and (2) and the theoretical plots in Figs. 2 and 3, were done using the 1D model described in^4^ in the steady-state regime ($\dot{\varphi } = 0$), extended to include external magnetic fields and neglecting the in-plane demagnetisation field:

$$ \left\{ {\begin{array}{ll} {\frac{\alpha v}{\Delta } = \gamma_{0} \left( {H_{Z} + \frac{\pi }{2}H_{SHE} \cos \varphi } \right)} \\ {\frac{v}{\Delta } = \gamma_{0} \frac{\pi }{2}\left( {\left( {H_{DMI} + H_{X} } \right)\sin \varphi + H_{Y} \cos \varphi } \right)} \\ \end{array} } \right. $$

with $H_{SHE} = \frac{\hbar }{2e}\frac{{\theta_{SHE} }}{{\mu_{0} M_{S} t}}J,\;H_{DMI} = \frac{D}{{\Delta \mu_{0} M_{S} }}$. In the absence of H_Z_, this yields $v = \frac{{\gamma_{0} \Delta }}{\alpha } \frac{\pi }{2}H_{SHE} \cos \varphi$, $\varphi = \tan \left( {\frac{{H_{SHE} /\alpha + H_{Y} }}{{H_{DMI} + H_{X} }}} \right)$. These equations can be used for ferrimagnets using the effective parameters^15,16,33^ as described above. The calculated plots in Fig. 3 are obtained using a constant ratio D/Δ obtained from the determination of *H*_DMI_ $\left( {\frac{D}{\Delta } = \mu_{0} M_{S} \left( T \right)H_{DMI} \left( T \right) = 2 \;{\text{kJ}}/{\text{m}}^{3} } \right)$, and the SOT factor $\frac{\hbar }{2e}\frac{{\theta_{SHE} }}{t}$ from the determination of *H*_DL_ (ℏ *θ*_SHE_/(2 *e t*) = *µ*_0_ *M*_S_(*T*) *H*_DL_(*T*)/*J* = 4.0 J·m^−3^/(GA·m^−2^)). The only parameter that is not experimentally determined, *α*(*T*), is approximated by an inverse linear law *α*(*T*) = 13.0 K/(*T* − *T*_AC_), chosen to best reproduce the shape of the experimental curves (see Figs. 2a and 3b). See supplementary materials for more results.

## Supplementary information

Supplementary Information.

## Data Availability

Raw data related to this paper may be requested from the authors.
