# Supplementary material for: Measurement of the tilt of a moving domain wall shows precession-free dynamics in compensated ferrimagnets
Source: Sci Rep. 2020 Oct 1;10:16292. doi: 10.1038/s41598-020-73049-5 (PMC7529739; doi:10.1038/s41598-020-73049-5)
Supplement: Supplementary file 1 — Supplementary Information. [file 41598_2020_73049_MOESM1_ESM.pdf]

Supplementary materials to

Measurement of the tilt of a moving domain wall shows precession-free dynamics in compensated ferrimagnets

E. Haltz[1], J. Sampaio[1], S. Krishnia[1], L. Berges[1], R. Weil[1] and A. Mougin[1]

[1] Université Paris-Saclay, CNRS, Laboratoire de Physique des Solides, 91405 Orsay, France

This supplementary contains additional data on the raw sample properties, mean field calculations, the extended equations used for analytical modelling with associated DW mobilities and a few additional experimental observations on DW propagation.

Contents

Section S1. Magnetization measurements
Section S2. Velocity measurements versus pulse duration
Section S3. Propagation under SOT with an in-plane field Hx for the determination of H_{DMI}
Section S4. Velocities curves
Section S5. Analytical model of DW velocity under SOT and field
Section S6. Sample parameters (measured and calculated)
Section S7. Crossing points versus current density and in-plane field

S1. Magnetization measurements

The magnetization of the virgin GdFeCo/Pt film has been measured over a larger temperature range than the one shown in fig. 1 of the article. Results are shown in Fig. S1, shifted by -31 K as described in the main text, along the mean field calculations.

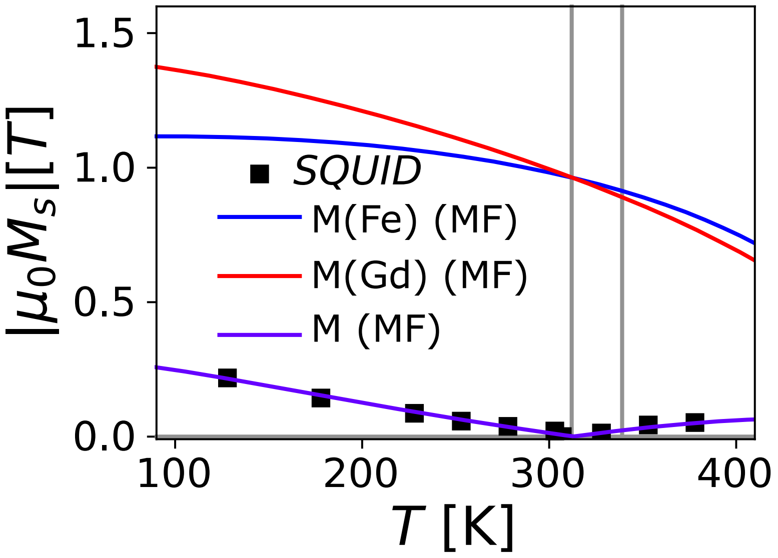

Fig. S1: Over a large temperature T range, mean-field (MF) calculations and measurements (SQUID) of |Ms|.

S2. Velocity measurements versus pulse duration

The linearity of the DW displacement with the number of pulses and with the pulse duration allows a reliable determination of the propagation velocity *v*. As shown in Fig. S2, the velocity does not depend on the pulse duration, nor on the number of pulses, whatever the current density.

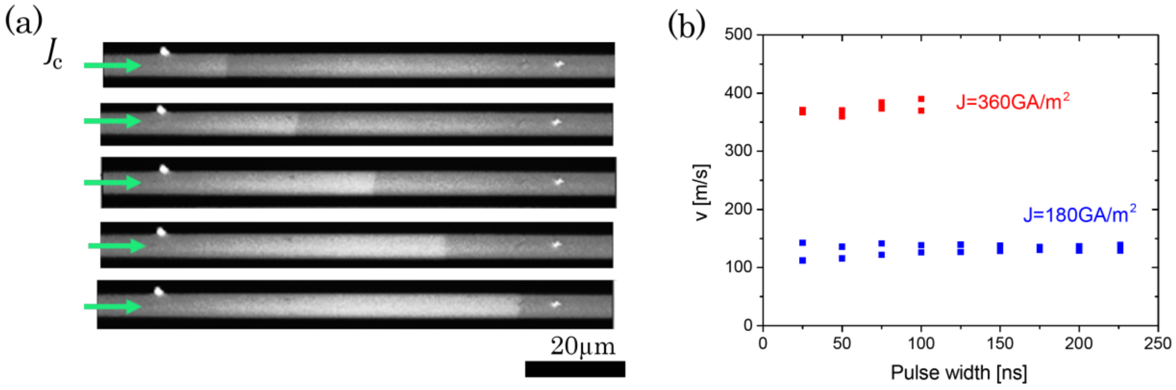

Fig. S2: (a) Kerr images of a DW driven by 300GA/m^2, 25 ns current pulses in the GdFeCo5nm/Pt7nm 10μm-track at temperature set-point of T_{SP} =300 K b) DW velocity versus pulse duration for two current densities indicated in the figure.

S3. Propagation under SOT with an in-plane field H_x for the determination of H_{DMI}

When an in-plane field is applied along the current flow (H_X) is larger than the effective DMI field, the DW propagation is reversed. We have measured this (fig. S3) and we have determined that μH_{DMI}=175mT at ~332K and 90mT at ~342K.

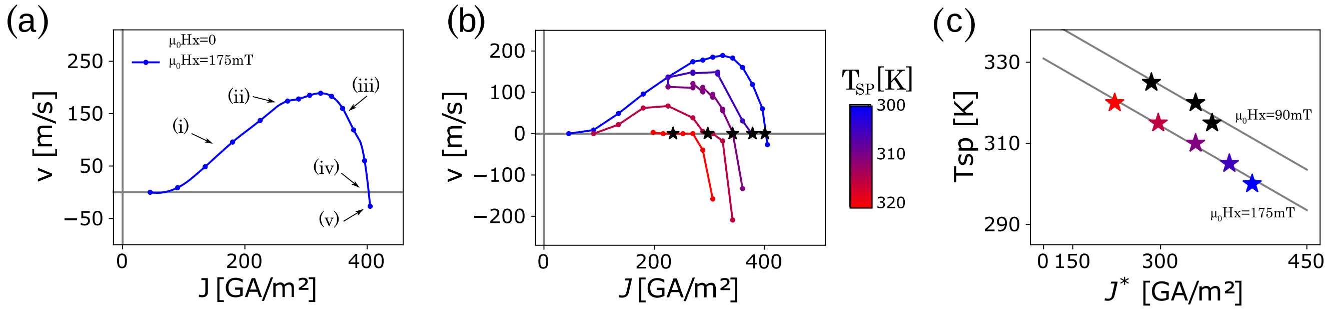

Fig S3. a) Domain wall propagation under SOT and in-plane field $\mu_0 H_X$= -175 mT, opposite to the DMI field at T_{SP} =300K. In grey, the DW velocity driven by SOT with no field. b) DW velocity versus J for different T_{SP} with $\mu_0 H_X$=-175mT. The reversal currents J*: v(J*)=0 are marked with stars. c) T_{SP} versus J* (quadratic horizontal scale) for two different in-plane fields. The extrapolated temperature at J*→0 gives the point when $H_{DMI} = H_X$.

S4. Velocities curves

DW motion occurs only above a threshold J of few tens of GA/m² that we attribute to DW pinning at defects, supported by the fact that the threshold J decreases when T_{SP} increases. Similar behaviour is often found in current-driven DWs, both in ferromagnets (ref. 5) and in ferrimagnets (refs. 19,20). In our wire, the threshold current is about 60 GA/m², a few times lower than in previous studies (refs. 19,20). Secondly, for a given T_{SP}, the velocity exhibits a non-monotonous behaviour (Fig S4a), while it is expected that the velocity is always increasing (refs. 4,5). The $v(J)$ measured at fixed T_{SP} (Fig. S4a) can be understood by considering the theoretical $v(J)$ curves for different T (Fig. S4a inset). Each measurement at fixed T_{SP} corresponds to a point in a curve $v(J)$ of progressively higher T as J increases, which produces a peak. In the mobility representation, the Joule heating

induces a simple horizontal shift between curves. For a given J, we observe a peak of mobility (marked by a star), up to 1.2 (m/s)/(GA/m²) (ten times higher than in previous reports (ref. 19,20)).

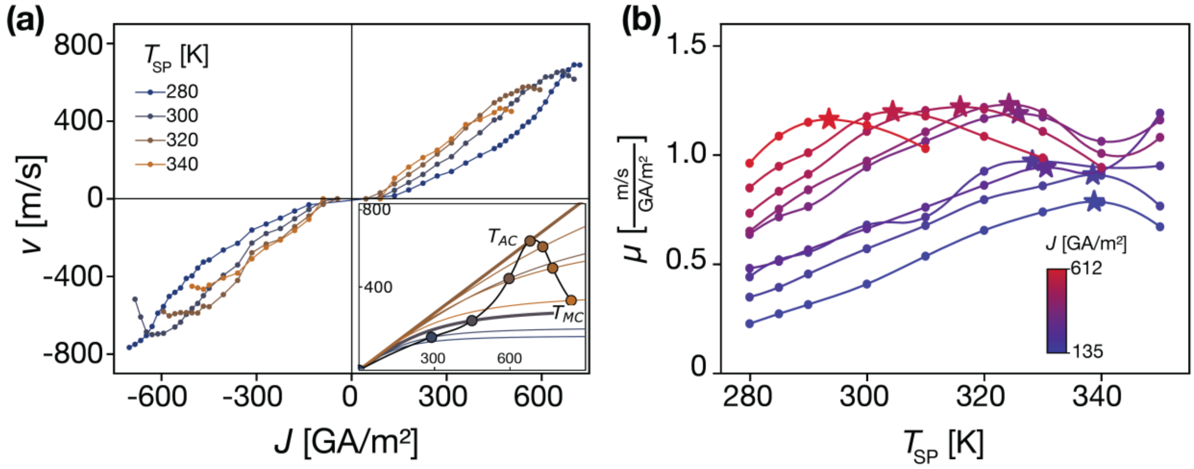

Fig S4. DW mobility versus current and temperature in a GdFeCo/Pt track. (a) DW velocity versus current density, $v(J)$, for different holder temperatures T_{SP}. **Inset:** theoretical $v(J)$ at constant track temperature T (blue to orange lines), and sketch of a $v(J)$ at constant T_{SP} considering Joule heating (coloured dots and black line). **(b)** DW mobility $\mu=v/J$ versus T_{SP} for some values of J. Maximum mobilities are marked by stars

S5. Analytical model of DW velocity under SOT and field

In the main text, Eqs. 1 & 2 and the theoretical plots in Fig. 3 were obtained using the 1D model described in (ref. 4) in the steady-state regime ($\dot{\varphi} = 0$), extended to include external magnetic fields and neglecting the in-plane demagnetisation field:

$$\begin{cases} \dfrac{\alpha v}{\Delta} = \gamma_0 \left(H_Z + \dfrac{\pi}{2} H_{SHE} \cos\varphi\right) \\ \dfrac{v}{\Delta} = \gamma_0 \, \dfrac{\pi}{2}\left((H_{DMI} + H_X)\sin\varphi + H_Y \cos\varphi\right) \end{cases} \Leftrightarrow \begin{cases} v = \dfrac{\gamma_0 \Delta}{\alpha}\left(H_Z + \dfrac{\pi}{2} H_{SHE}\cos\varphi\right) \\ H_Z + (H_{SHE} - \alpha\, H_Y)\dfrac{\pi}{2}\cos\varphi = \alpha\, \dfrac{\pi}{2}(H_{DMI} + H_X)\sin\varphi \end{cases}$$

with $H_{SHE} = \frac{\hbar}{2e}\frac{\theta_{SHE}}{\mu_0 M_S\, t} J$, $H_{DMI} = \frac{D}{\Delta\, \mu_0 M_S}$.

In the absence of H_Z, this yields:

$$v = \frac{\gamma_0 \Delta}{\alpha}\frac{\pi}{2} H_{SHE}\cos\varphi, \qquad \varphi = \arctan\left(\frac{H_{SHE}/\alpha + H_Y}{H_{DMI} + H_X}\right)$$

With H_Z smaller than the Walker field, this yields:

$$v = \frac{\gamma_0 \Delta}{\alpha}\frac{\left((H_{DMI}+H_X)^2 + H_Y^2\right)H_Z\alpha^2 + H_{SHE}\left(H_Y H_Z \alpha + \alpha(H_{DMI}+H_X)\sqrt{\frac{\pi^2}{4}A - H_Z^2}\right)}{A},$$

with $A = H_{SHE}^2 + 2\alpha H_{SHE} H_Y + \alpha^2\left((H_{DMI} + H_X)^2 + H_Y^2\right)$. These equations can be used for ferrimagnets using the effective parameters (refs. 15,16,33) as described above and in ref. 32. The calculated plots in Fig. 3 are obtained using a constant ratio D/Δ obtained from the determination of H_{DMI} ($\frac{D}{\Delta} = \mu_0 M_S(T) H_{DMI}(T) = 2\ kJ/m^3$), and the SOT factor $\frac{\hbar}{2e}\frac{\theta_{SHE}}{t}$ from the determination of H_{SHE} ($\frac{\hbar}{2e}\frac{\theta_{SHE}}{t} = \mu_0 M_S(T) H_{SHE}(T)/_J = 4.0\ \frac{J/m^3}{GA/m^2}$). Only the parameters , α(T) and Δ are not experimentally determined, and were chosen to best

reproduce the shape of the experimental curves. α(T) is approximated by an inverse linear law $\alpha(T) = \frac{13\ \mathrm{K}}{T - T_{AC}}$, (see Fig. 3), and $\Delta = 10\mathrm{nm}$.

S6. Sample parameters (measured and calculated)

Several sample parameters are shown in Fig. S5 versus temperature. In the effective parameter approach (see references in the main text) $M_S = M_1 - M_2$, $L_S = L_1 - L_2$, $L_i = M_i/\gamma_1$, and $\gamma_{0\ eff} = \mu_0 M_S / L_S$

The net magnetization and the gyromagnetic ratio γ_{eff} change sign at T_{MC} whereas the angular momentum and the effective damping α_{eff} change sign at T_{AC}. The static parameters (H_{DMI} and H_{SHE}) change sign and diverge at T_{MC} whereas the dynamic ones (γ_{0eff} and α_{eff}) change sign and diverge at T_{AC}. SOT driven domain wall mobility shows a peak at T_{AC}.

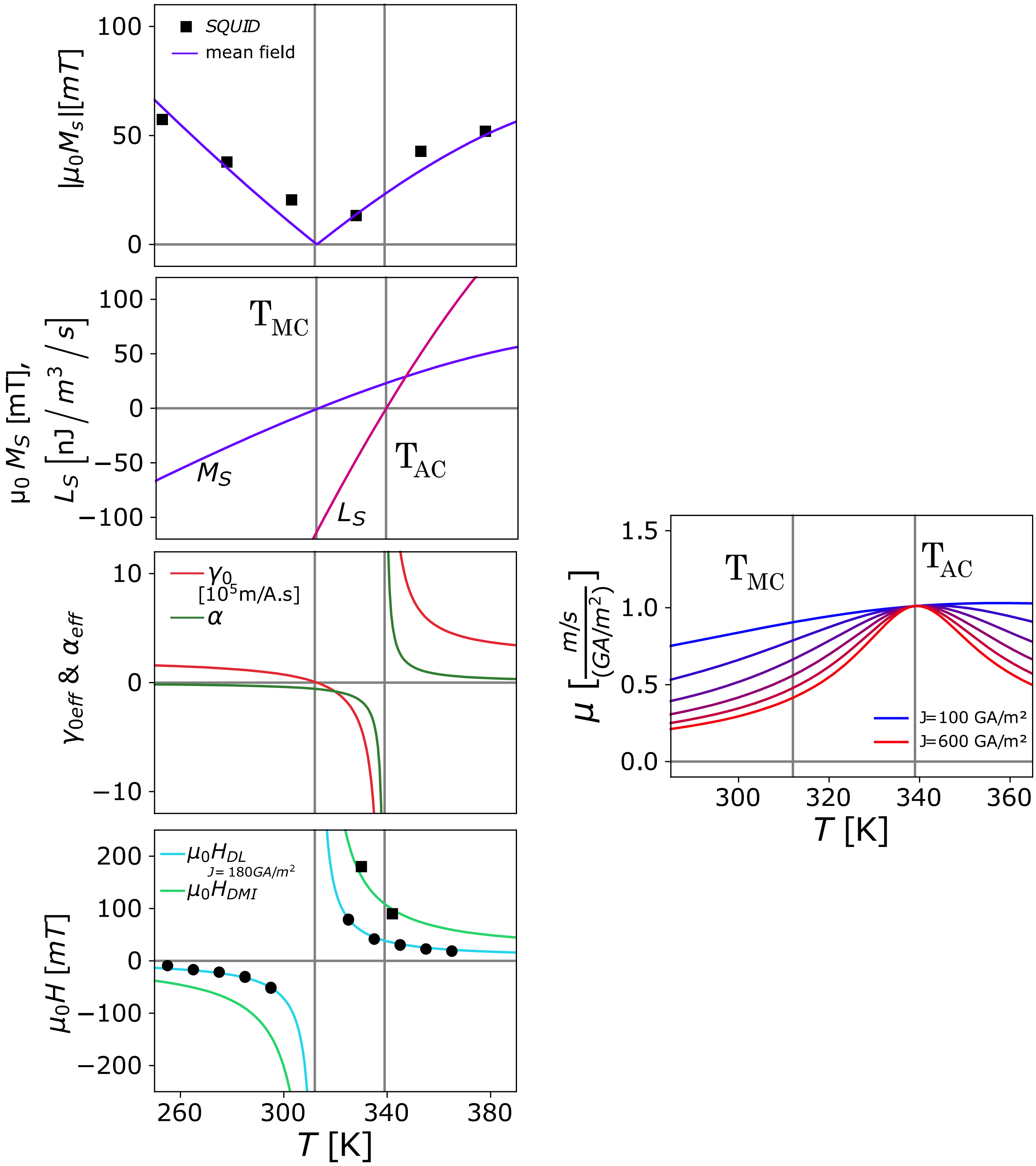

Fig S5: From top to bottom, left to right, over a temperature range close to that of experiments: Mean-field calculations and measurements of |Ms|; net magnetization and angular momentum; effective gyromagnetic ratio $\gamma_{0\ eff}$ (10^5 m/As) and damping α_{eff}; H_{DMI} and H_{DL} (for J=180 GA/m^2); calculated mobilities for several current densities.

S7. Crossing points versus current density and in-plane field

We measured the DW velocity v versus T_{SP} with an applied in-plane field H_Y perpendicular to the current flow. In the main text, velocities are shown for J=360 GA/m^2; and here Fig. S6 shows other values of J.

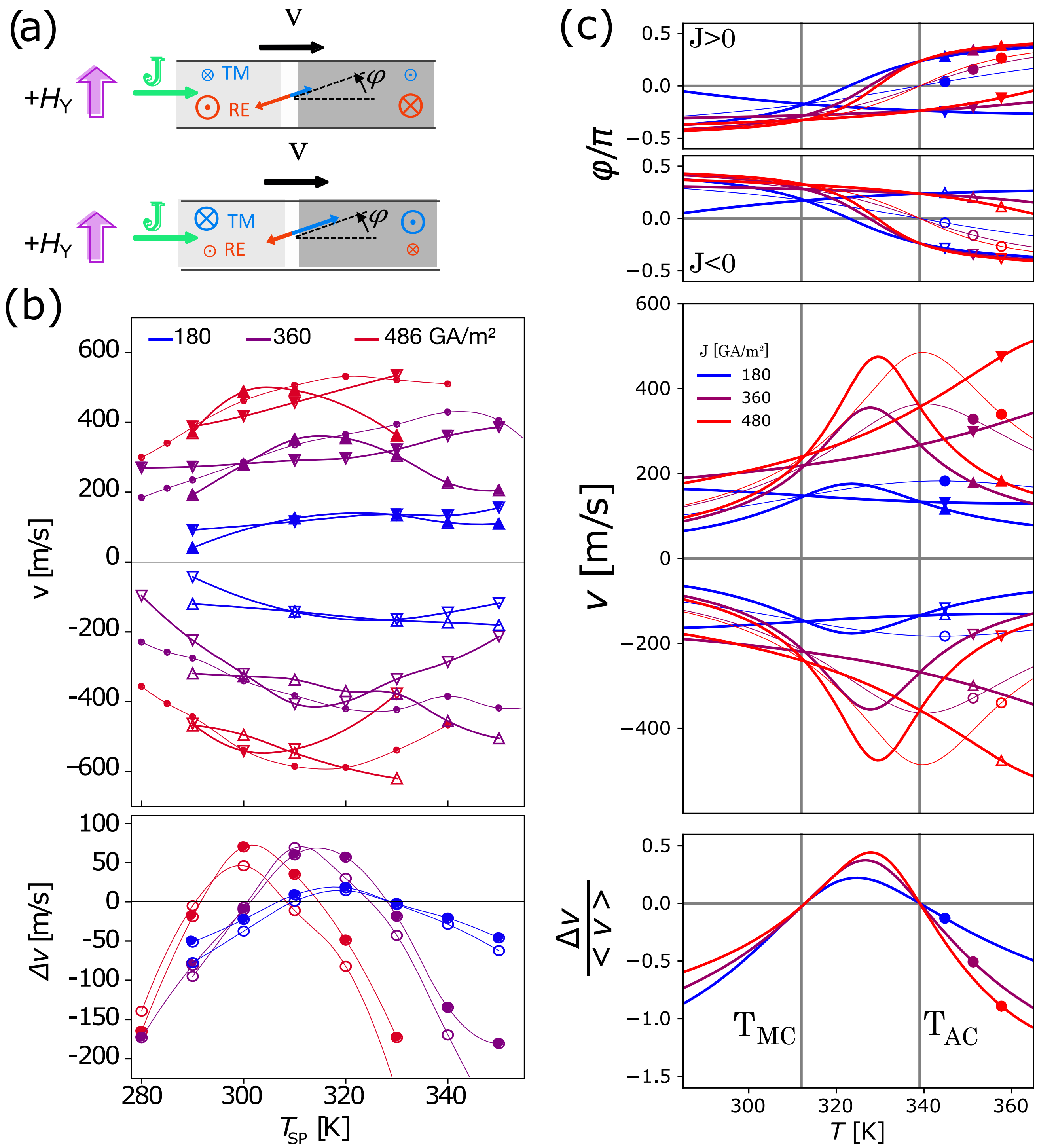

Fig. S6: (a) Sketch of a SOT-driven DW under H_Y. The red and blue arrows correspond to RE and TM, respectively below and above T_{MC}. The size of the arrows represents their relative magnitude. The grayscale corresponds to the domain Kerr contrast while the DW is depicted in white. The angle of the DW magnetisation is given by φ. The purple arrows represent H_Y, and the green ones the current. (**b**) DW velocity v versus sample holder temperature T_{SP} with $H_Y = \pm 90$ mT (▲,▼) or 0 mT (●), for ± J (top and bottom parts) and velocity difference $\Delta v(T_{SP}) \equiv$v(J,+H_Y)-v(J,-H_Y) for the same H_Y and J. **(c)** Calculated DW angle φ, DW velocity $v \propto \cos\varphi$ using the equations in the text and velocity difference Δv versus track temperature T.

When an in-plane field transversal to the current direction is applied in DW propagation experiments, two crossing points, $T_{SP,1}$ and $T_{SP,2}$, are observed where $v(T_{SP,i}, +H_Y) = v(T_{SP,i}, -H_Y)$. Fig S7 shows the difference measured between the temperatures of these two crossing-points.

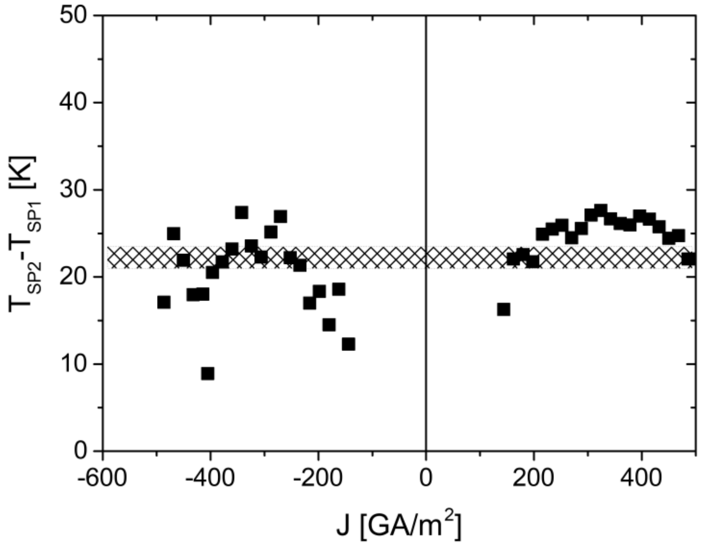

FIG S7: $T_{SP,2} - T_{SP,1}$ vs J for SOT driven DW with an in-plane field.

Fig.S8 (a) shows the mobility of the DW driven by SOT under positive, negative and zero transverse fields as a function of temperature for different H_Y and J. The temperature T was obtained using the Joule heating law given in the main text. Similarly to the Fig. 2A of the main text, for every H_Y, a crossing point is obtained where μ(+H_Y)=μ(-H_Y) (as indicated by the grey point). The temperature of the crossing point is the same whatever the H_Y (T_{AC}). Fig.S8 (b) shows the mobility associated to the crossing point for each transverse field. The black line corresponds to the fit of this mobility by using a simplified version of eq. 2 : $\mu(H_Y) = A\cos(\arctan(B + C\ H_Y))$. The black points correspond to the values of the mobility coming from the fit without transverse field: $\mu\ (H_Y = 0) = A\cos(\arctan B)$ (with A and B coming from the above fit). It is possible to verify that this feature is captured by the eq. 2 and that the obtained values of $\mu\ (H_Y = 0)$ corresponds to the experimental value of μ(H_Y=0) at the crossing point temperature.

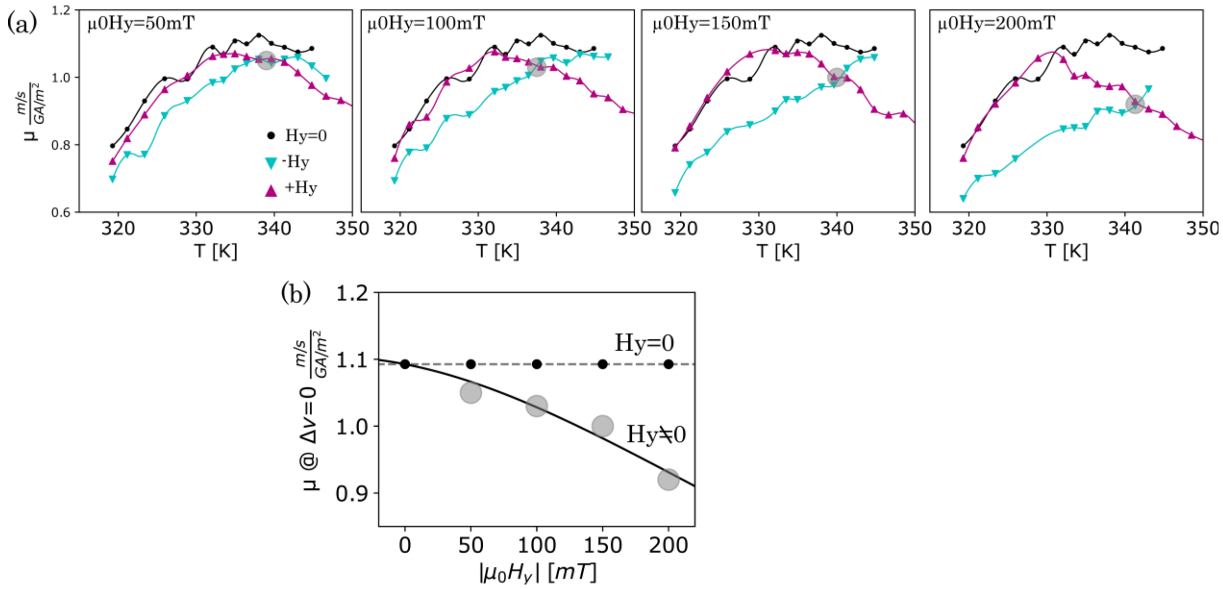

FIG S8. (a) DW mobility (μ) versus T for different H_Y at T_{SP}=315 K and different J. **(b)** Mobility at the crossing point versus H_Y.

Spurious external fields have an impact only along the y direction, as confirmed in the calculated $\Delta v/\langle v\rangle$ with an offset external field (Fig. S9), which may increase the data dispersion at T_{AC}.

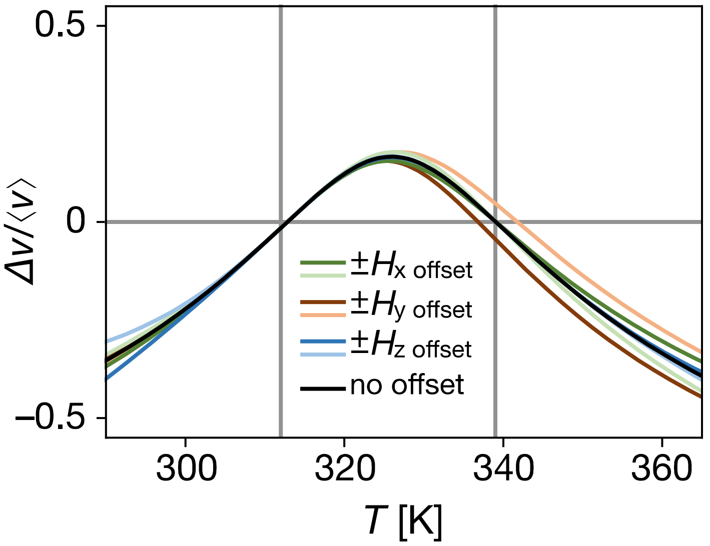

Fig. S9. Theoretical $\Delta v/\langle v\rangle$ with an offset bias field of 10 mT along x, y or z ($|H_Y|$ = 90 mT and J=360 GA/m²) .
